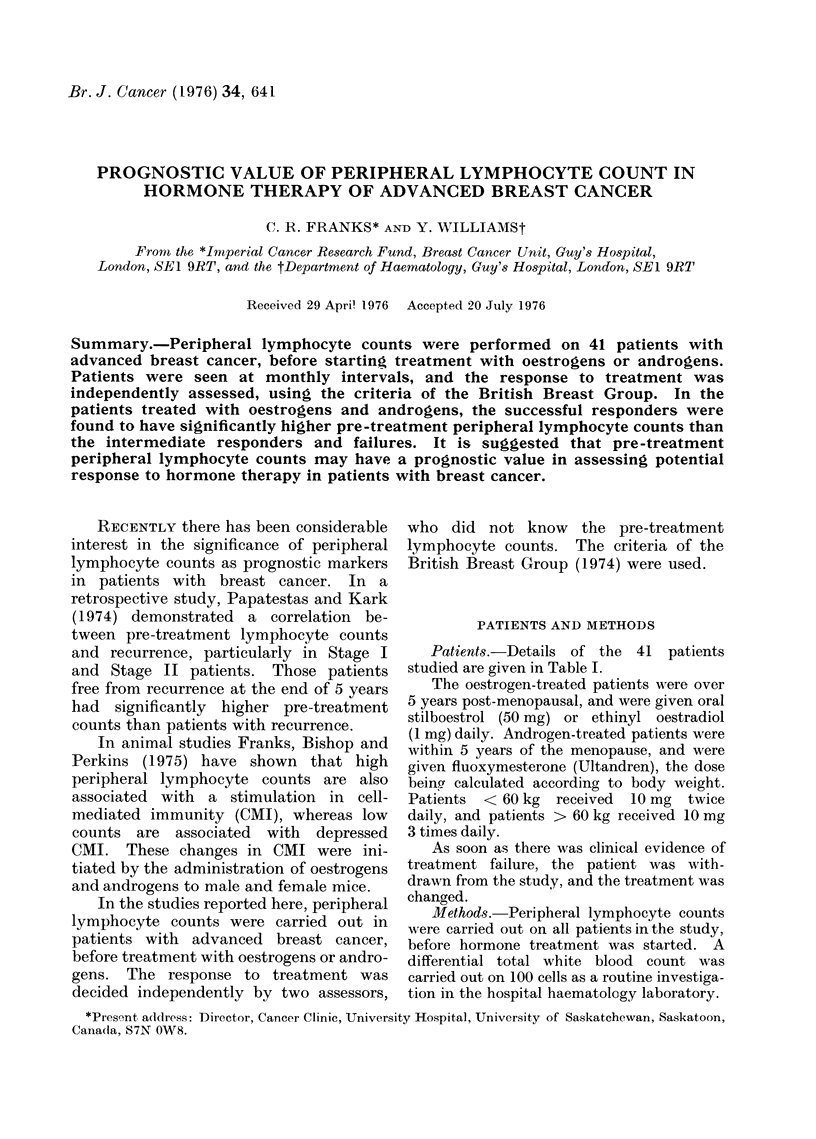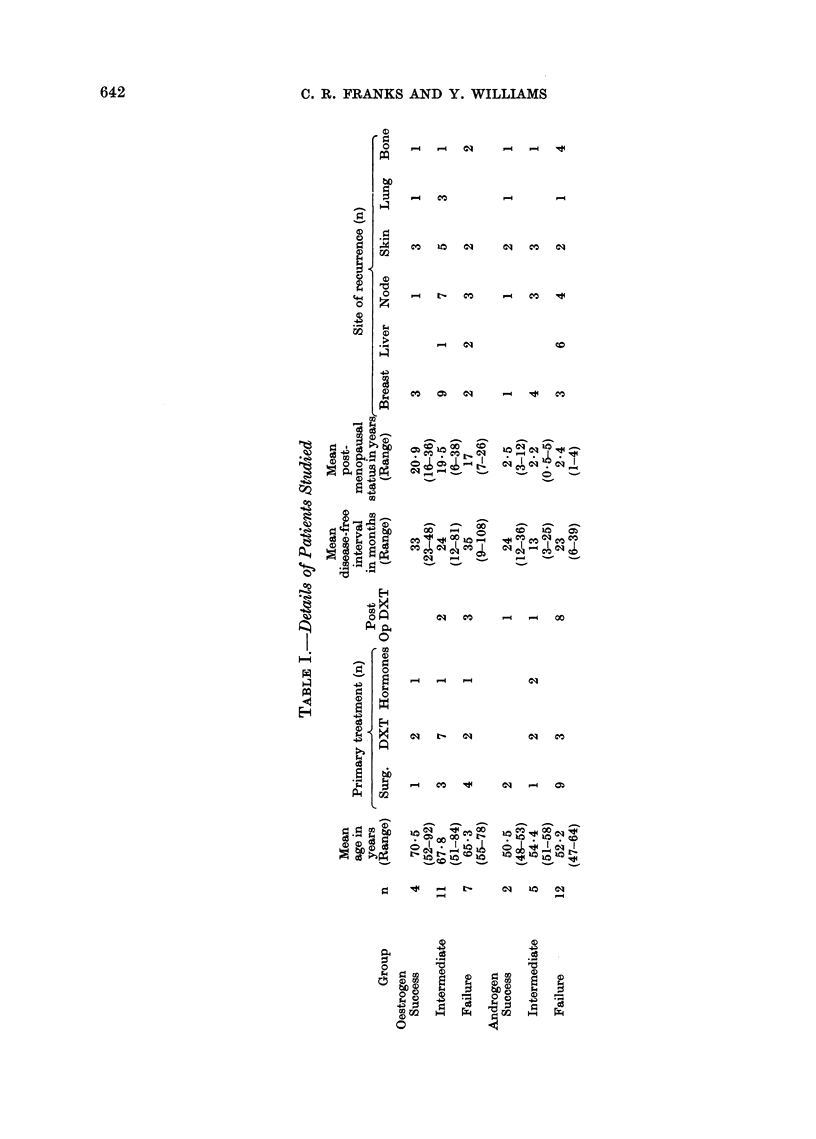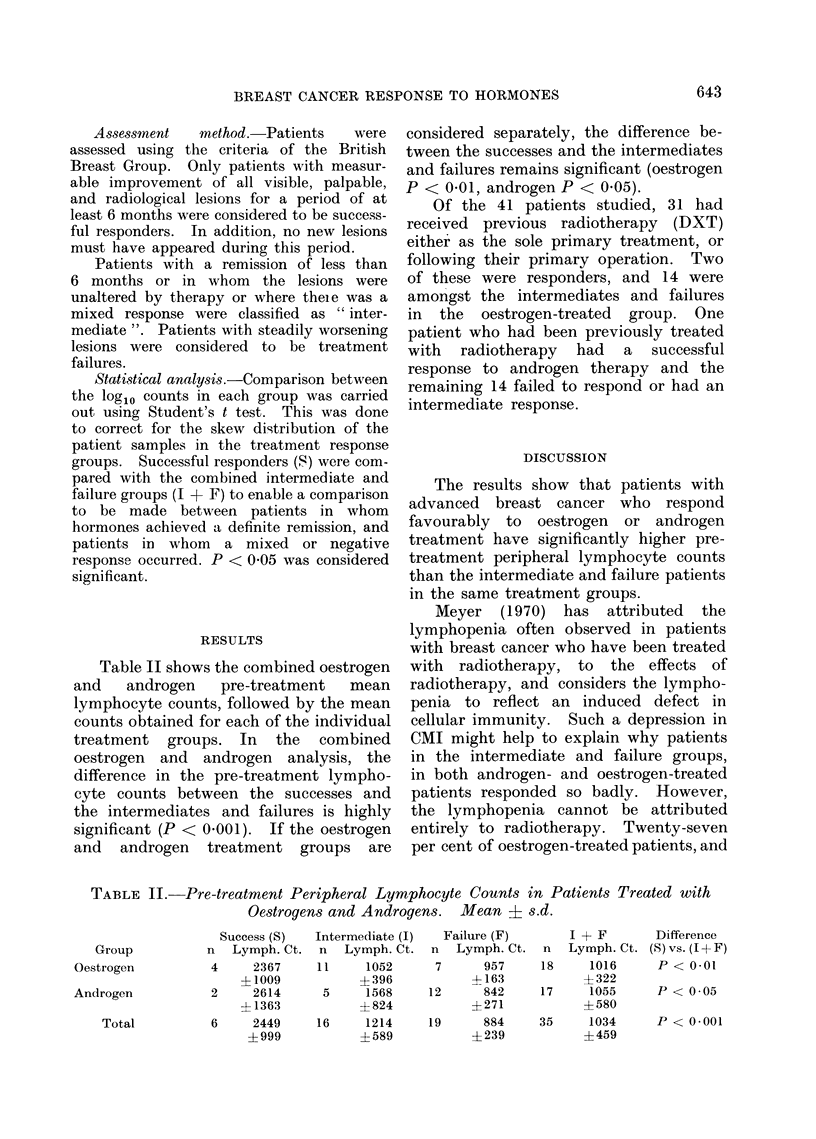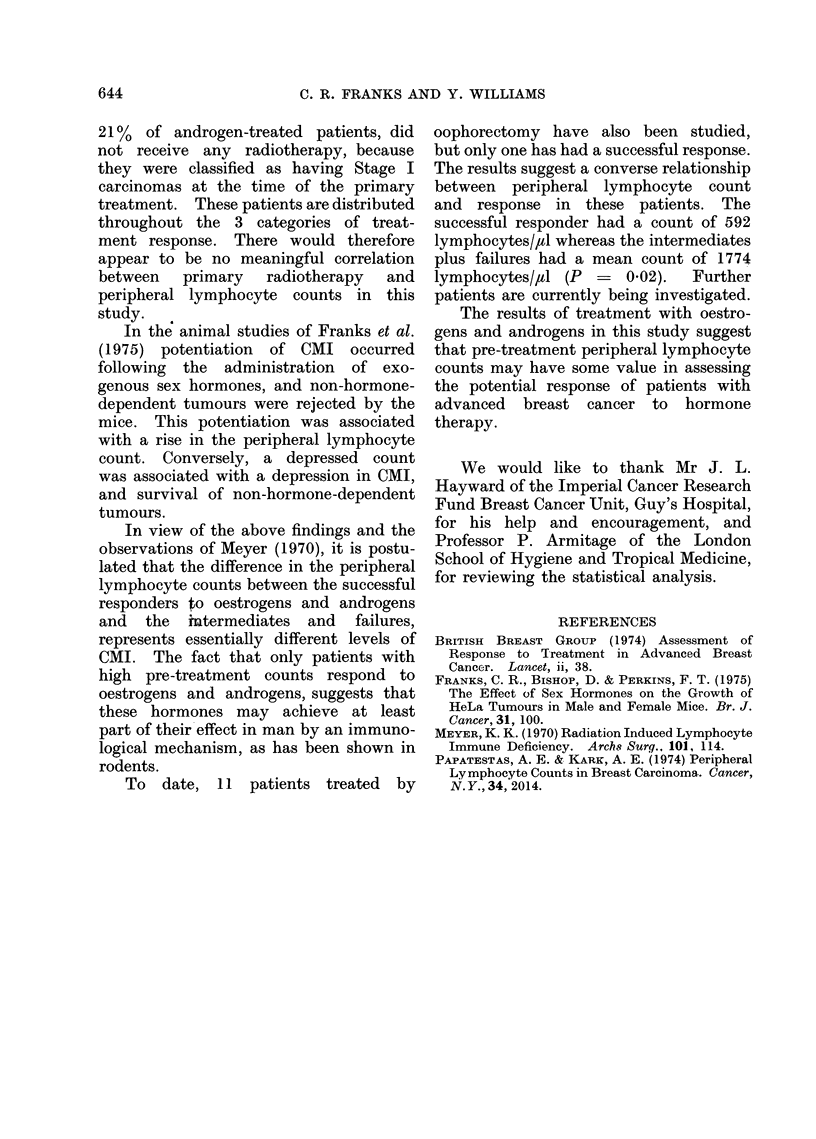# Prognostic value of peripheral lymphocyte count in hormone therapy of advanced breast cancer.

**DOI:** 10.1038/bjc.1976.225

**Published:** 1976-12

**Authors:** C. R. Franks, Y. Williams

## Abstract

Peripheral lymphocyte counts were performed on 41 patients with advanced breast cancer, before starting treatment with oestrogens or androgens. Patients were seen at monthly intervals, and the response to treatment was independently assessed, using the criteria of the British Breast Group. In the patients treated with oestrogens and androgens, the successful responders were found to have significantly higher pre-treatment peripheral lymphocyte counts than the intermediate responders and failures. It is suggested that pre-treatment peripheral lymphocyte counts may have a prognostic value in assessing potential response to hormone therapy in patients with breast cancer.


*Br. J. Cancer* (1976) **34**, 641

### PROGNOSTIC VALUE OF PERIPHERAL LYMPHOCYTE COUNT IN HORMONE THERAPY OF ADVANCED BREAST CANCER

C. R. FRANKS* AND Y. WILLIAMS†

*From the *Imperial Cancer Research Fund, Breast Cancer Unit, Guy's Hospital, London, SE1 9RT, and the †Department of Haematology, Guy's Hospital, London, SE1 9RT*

Received 29 April 1976 Accepted 20 July 1976

**Summary.—Peripheral lymphocyte counts were performed on 41 patients with advanced breast cancer, before starting treatment with oestrogens or androgens. Patients were seen at monthly intervals, and the response to treatment was independently assessed, using the criteria of the British Breast Group. In the patients treated with oestrogens and androgens, the successful responders were found to have significantly higher pre-treatment peripheral lymphocyte counts than the intermediate responders and failures. It is suggested that pre-treatment peripheral lymphocyte counts may have a prognostic value in assessing potential response to hormone therapy in patients with breast cancer.**

RECENTLY there has been considerable interest in the significance of peripheral lymphocyte counts as prognostic markers in patients with breast cancer. In a retrospective study, Papatestas and Kark (1974) demonstrated a correlation between pre-treatment lymphocyte counts and recurrence, particularly in Stage I and Stage II patients. Those patients free from recurrence at the end of 5 years had significantly higher pre-treatment counts than patients with recurrence.

In animal studies Franks, Bishop and Perkins (1975) have shown that high peripheral lymphocyte counts are also associated with a stimulation in cell-mediated immunity (CMI), whereas low counts are associated with depressed CMI. These changes in CMI were initiated by the administration of oestrogens and androgens to male and female mice.

In the studies reported here, peripheral lymphocyte counts were carried out in patients with advanced breast cancer, before treatment with oestrogens or androgens. The response to treatment was decided independently by two assessors, who did not know the pre-treatment lymphocyte counts. The criteria of the British Breast Group (1974) were used.

### PATIENTS AND METHODS

*Patients.*—Details of the 41 patients studied are given in Table I.

The oestrogen-treated patients were over 5 years post-menopausal, and were given oral stilboestrol (50 mg) or ethinyl oestradiol (1 mg) daily. Androgen-treated patients were within 5 years of the menopause, and were given fluoxymesterone (Ultandren), the dose being calculated according to body weight. Patients $< 60$ kg received 10 mg twice daily, and patients $> 60$ kg received 10 mg 3 times daily.

As soon as there was clinical evidence of treatment failure, the patient was withdrawn from the study, and the treatment was changed.

*Methods.*—Peripheral lymphocyte counts were carried out on all patients in the study, before hormone treatment was started. A differential total white blood count was carried out on 100 cells as a routine investigation in the hospital haematology laboratory.

*Present address: Director, Cancer Clinic, University Hospital, University of Saskatchewan, Saskatoon, Canada, S7N 0W8.

TABLE I.—*Details of Patients Studied*

| Group | n | Mean age in years (Range) | Primary treatment (n) Surg. | DXT | Hormones | Post Op DXT | Mean disease-free interval in months (Range) | Mean post-menopausal status in years (Range) | Site of recurrence (n) Breast | Liver | Node | Skin | Lung | Bone |
|---|---|---|---|---|---|---|---|---|---|---|---|---|---|---|
| Oestrogen | | | | | | | | | | | | | | |
| Success | 4 | 70·5 (52–92) | 1 | 2 | 1 | | 33 (23–48) | 20·9 (16–36) | 3 | | 1 | 3 | 1 | 1 |
| Intermediate | 11 | 67·8 (51–84) | 3 | 7 | 1 | 2 | 24 (12–81) | 19·5 (6–38) | 9 | 1 | 7 | 5 | 3 | 1 |
| Failure | 7 | 65·3 (55–78) | 4 | 2 | 1 | 3 | 35 (9–108) | 17 (7–26) | 2 | 2 | 3 | 2 | | 2 |
| Androgen | | | | | | | | | | | | | | |
| Success | 2 | 50·5 (48–53) | 2 | | | 1 | 24 (12–36) | 2·5 (3–12) | 1 | | 1 | 2 | 1 | 1 |
| Intermediate | 5 | 54·4 (51–58) | 1 | 2 | 2 | 1 | 13 (3–25) | 2·2 (0·5–5) | 4 | | 3 | 3 | | 1 |
| Failure | 12 | 52·2 (47–64) | 9 | 3 | | 8 | 23 (6–39) | 2·4 (1–4) | 3 | 6 | 4 | 2 | 1 | 4 |

*Assessment method.*—Patients were assessed using the criteria of the British Breast Group. Only patients with measurable improvement of all visible, palpable, and radiological lesions for a period of at least 6 months were considered to be successful responders. In addition, no new lesions must have appeared during this period.

Patients with a remission of less than 6 months or in whom the lesions were unaltered by therapy or where theie was a mixed response were classified as " intermediate ". Patients with steadily worsening lesions were considered to be treatment failures.

*Statistical analysis.*—Comparison between the $\log_{10}$ counts in each group was carried out using Student's $t$ test. This was done to correct for the skew distribution of the patient samples in the treatment response groups. Successful responders (S) were compared with the combined intermediate and failure groups (I + F) to enable a comparison to be made between patients in whom hormones achieved a definite remission, and patients in whom a mixed or negative response occurred. $P < 0{\cdot}05$ was considered significant.

### RESULTS

Table II shows the combined oestrogen and androgen pre-treatment mean lymphocyte counts, followed by the mean counts obtained for each of the individual treatment groups. In the combined oestrogen and androgen analysis, the difference in the pre-treatment lymphocyte counts between the successes and the intermediates and failures is highly significant ($P < 0{\cdot}001$). If the oestrogen and androgen treatment groups are considered separately, the difference between the successes and the intermediates and failures remains significant (oestrogen $P < 0{\cdot}01$, androgen $P < 0{\cdot}05$).

Of the 41 patients studied, 31 had received previous radiotherapy (DXT) either as the sole primary treatment, or following their primary operation. Two of these were responders, and 14 were amongst the intermediates and failures in the oestrogen-treated group. One patient who had been previously treated with radiotherapy had a successful response to androgen therapy and the remaining 14 failed to respond or had an intermediate response.

### DISCUSSION

The results show that patients with advanced breast cancer who respond favourably to oestrogen or androgen treatment have significantly higher pre-treatment peripheral lymphocyte counts than the intermediate and failure patients in the same treatment groups.

Meyer (1970) has attributed the lymphopenia often observed in patients with breast cancer who have been treated with radiotherapy, to the effects of radiotherapy, and considers the lymphopenia to reflect an induced defect in cellular immunity. Such a depression in CMI might help to explain why patients in the intermediate and failure groups, in both androgen- and oestrogen-treated patients responded so badly. However, the lymphopenia cannot be attributed entirely to radiotherapy. Twenty-seven per cent of oestrogen-treated patients, and

TABLE II.—*Pre-treatment Peripheral Lymphocyte Counts in Patients Treated with Oestrogens and Androgens. Mean ± s.d.*

| Group | Success (S) n | Success (S) Lymph. Ct. | Intermediate (I) n | Intermediate (I) Lymph. Ct. | Failure (F) n | Failure (F) Lymph. Ct. | I + F n | I + F Lymph. Ct. | Difference (S) vs. (I+F) |
|---|---|---|---|---|---|---|---|---|---|
| Oestrogen | 4 | 2367 ±1009 | 11 | 1052 ±396 | 7 | 957 ±163 | 18 | 1016 ±322 | $P < 0{\cdot}01$ |
| Androgen | 2 | 2614 ±1363 | 5 | 1568 ±824 | 12 | 842 ±271 | 17 | 1055 ±580 | $P < 0{\cdot}05$ |
| Total | 6 | 2449 ±999 | 16 | 1214 ±589 | 19 | 884 ±239 | 35 | 1034 ±459 | $P < 0{\cdot}001$ |

21% of androgen-treated patients, did not receive any radiotherapy, because they were classified as having Stage I carcinomas at the time of the primary treatment. These patients are distributed throughout the 3 categories of treatment response. There would therefore appear to be no meaningful correlation between primary radiotherapy and peripheral lymphocyte counts in this study.

In the animal studies of Franks *et al.* (1975) potentiation of CMI occurred following the administration of exogenous sex hormones, and non-hormone-dependent tumours were rejected by the mice. This potentiation was associated with a rise in the peripheral lymphocyte count. Conversely, a depressed count was associated with a depression in CMI, and survival of non-hormone-dependent tumours.

In view of the above findings and the observations of Meyer (1970), it is postulated that the difference in the peripheral lymphocyte counts between the successful responders to oestrogens and androgens and the intermediates and failures, represents essentially different levels of CMI. The fact that only patients with high pre-treatment counts respond to oestrogens and androgens, suggests that these hormones may achieve at least part of their effect in man by an immunological mechanism, as has been shown in rodents.

To date, 11 patients treated by oophorectomy have also been studied, but only one has had a successful response. The results suggest a converse relationship between peripheral lymphocyte count and response in these patients. The successful responder had a count of 592 lymphocytes/$\mu$l whereas the intermediates plus failures had a mean count of 1774 lymphocytes/$\mu$l ($P = 0{\cdot}02$). Further patients are currently being investigated.

The results of treatment with oestrogens and androgens in this study suggest that pre-treatment peripheral lymphocyte counts may have some value in assessing the potential response of patients with advanced breast cancer to hormone therapy.

We would like to thank Mr J. L. Hayward of the Imperial Cancer Research Fund Breast Cancer Unit, Guy's Hospital, for his help and encouragement, and Professor P. Armitage of the London School of Hygiene and Tropical Medicine, for reviewing the statistical analysis.

### REFERENCES

British Breast Group (1974) Assessment of Response to Treatment in Advanced Breast Cancer. *Lancet*, ii, 38.

Franks, C. R., Bishop, D. & Perkins, F. T. (1975) The Effect of Sex Hormones on the Growth of HeLa Tumours in Male and Female Mice. *Br. J. Cancer*, **31**, 100.

Meyer, K. K. (1970) Radiation Induced Lymphocyte Immune Deficiency. *Archs Surg.*, **101**, 114.

Papatestas, A. E. & Kark, A. E. (1974) Peripheral Lymphocyte Counts in Breast Carcinoma. *Cancer, N.Y.*, **34**, 2014.